# Transactions of the American Med. Association, Instituted 1847

**Published:** 1850-07

**Authors:** 


### ARTICLE I.

*Transactions of the American Medical Association, Instituted* 1847. Vol. 2, pp. 956, 8vo. Philadelphia: Printed for the Asssociation, by T. K. & P. G. Collins, 1848.

In continuing a notice of the contents of this volume, which was commenced in the last preceding number, we come next in order to the Report of the Standing Committee on Medical Education.

The duties of this committee are defined in the following extract, viz :—

"1st. An examination into the general condition of Medical Education in the United States, in comparison with the state of medical education in other enlightened nations; noticing, as occasion may call for, the courses of instruction, the practical requirements for graduation, the modes of examination for conferring degrees, and the number of pupils and of graduates at the several medical institutions in the United States.

"2d. The requirements of the United States Army and Navy Boards of Medical Examiners.

"3d. The legal requirements exacted of medical practitioners in the several States of the Union.

"4th. Such measures, prospective or established, in reference to medical education, and the reputable standing of the profession, as may be deemed worthy of special consideration."

The Chairman of the Committee was Dr. F. Campbell Stewart, of New York city.

The report occupies about one hundred pages of the Transactions, and is one of the ablest in the present volume. It gives a satisfactory, though brief, account of the rules, regulations, and requirements in force in the principal European institutions for medical instruction, and the conferring of degrees; following which is a more extended and equally judicious summary of the general condition of medical institutions and education in our own country. This part of the Report, not only embodies all the facts necessary to a fair understanding of the systems of medical education, at home and abroad, but it embraces also many extracts from the correspondence of the Chairman of the Committee, showing the views and opinions of many eminent members of the profession, and especially those connected with our colleges. From this report it appears that the shortest time allowed for the study of medicine in any of the countries of Europe, is *four* years, and the least time spent in attendance on lectures, during the period of study, is an aggregate of *two years*, or four courses of six months each,—while in this country the longest time required is three years, including two courses of college instruction of from fourteen weeks to five months each; or less than half of the college instruction required in Great Britain, and only about one-third of that given in all the continental countries. The following paragraphs taken from the report, contain sentiments worthy of serious consideration, viz:—

"By an examination of the details given in the preceding pages, it will be perceived that the system of instruction in medicine, in vogue in European countries is much more perfect and satisfactory than with us.

"The time required to be devoted in study is not only longer, but a preparatory education is, in all instances insisted upon, as being absolutely essential; and young men, before commencing their medical studies, must furnish abundant and satisfactory evidence of their having attained a given, and by no means small, amount of academic learning. It is wisely considered that, without preliminary training and knowledge in the several departments of general science, their minds are not properly prepared for receiving instruction in the high and complicated branches of professional education.

"The subjects taught in Europe are more numerous, and a much greater proportion of time is devoted to their study than s allowed in the United States. They are so disposed, also, that they follow each other in a regular consecutive order. The student is thus enabled to prepare himself on a given number of subjects, by close application to their details, in a reasonable period of time; after which he is examined, and, if successful, his mind being relieved of a portion of care and anxiety, he is better prepared to commence and prosecute the study of a new series, upon which he is likewise, in turn, examined, and which he dismisses, for the time being, from his thoughts. How infinitely superior is this course to that which compels him to burden his memory, and toil during the whole period of his attendance on lectures, to keep pace with his preceptors in their endeavors to impart to him instruction on a multitude of subjects which are crowded together, and a knowledge of all of which must be obtained in the very short space of time allowed by most of our colleges as the period in which their courses are comprised. * * * * The division of the scholastic year into summer and winter courses, which is practised at all of the institutions abroad, is a judicious one, and worthy of our imitation. * * *

"The advantages of having Professors made independent of their students, in being paid for their services wholly, or in part, by the State, are self-evident. This course is pursued everywhere in Europe, except in Great Britain, where teachers are, as in this country, paid by fees alone."

Under the head of "Recommendations for the Improvement of the Profession," the report insists strongly, and justly, too, on the necessity of proper attention to preliminary education, giving a plan by which it may be insured; of true Hospital or Clinical instruction; of more rigid public examinations for the Degree of M. D.; of lengthening the lecture terms, and dividing the subjects taught into two series, that they may be pursued in consecutive order; and of establishing in each State a Board of State Censors entirely unconnected with teaching, whose duty it shall be to examine rigidly, all candidates for admission into the ranks of the profession as practitioners in the several States, whether they are graduates of a medical college or not. In reference to this last recommendation, the report says:—

"We think that no material and lasting good can arise from an elevation of a standard of requirements by one or two colleges alone. The only manner in which the desired end could be obtained, would be by the co-operation of all the colleges, in the establishment of a uniform curriculum ;—and is this practicable ? can it be expected that rival institutions, possessing a great diversity of interests, and unequal facilities for teaching, will unite in a general scheme, which would have the effect of concentrating students at such colleges as are in reality best prepared to impart instruction ? We unhesitatingly answer in the negative ; *it is useless even to ask it.*

"But let each State of our Union authorize the establishment of a Board of disinterested Examiners, who shall have no direct interest in view, and the obtaining of whose certificate shall be always obligatory, prior to permitting an individual to engage in practice, and a direct check is at once imposed, which will make it to the interest of the schools to raise their standard to the level of that established by the licensing Boards, and a fair and just rivalry will then commence between the colleges, *as to which shall impart the requisite amount of instruction in the shortest space of time, and with the least expense to the student.*"

In another place the report adds :—

"The plan of establishing independent Examining and Licensing Boards, is, as you are well aware, neither original with us, nor a new thing in the United States. It has been so often and so freely discussed, that all of you are familiar with the subject, and its successful introduction into some of the States, where it is now in force, shows that it is both practicable and beneficial to the people and the profession. * * * *. It has also been shown, in another part of this report, that the system is in vogue, and is found to work well in many parts of Europe, where its adoption has been attended with the most satisfactory and beneficial results."

These sentiments are the dictates of sound common sense. The idea, so much talked of by some, of getting our forty medical colleges to unite in the *practical* adoption of an elevated curriculum of study and requirements, or of a uniform rate of fees, is simply absurd. Just so long as their interests are local, diverse, and selfish, there will be no other practical concert of action on these topics, than that which is sometimes brought about between the extreme advocate of southern slavery and the rank northern abolitionist in Congress, viz: a union to oppose some practical measure, not satisfactory to either extreme. The whole report is an extremely interesting and important one, and is alone worth the price of the whole volume. Appended to it, are two papers; one from the medical Faculty of Harvard College, opposed to the extension of the lecture term beyond *four* months, the other from a Select Committee, appointed by the Association to present the reasons, in full, in favor of lengthening the time of college instruction to six months. The latter paper is drawn up with ability, and presents facts and ideas which ought to be well considered by every member of the profession. If the following sentiments were generally remembered and acted upon, there would be less difficulty in advancing the common interests of the profession. This sub-report says:

"The subject of medical education is not limited to a mere consideration of *the interests of students and schools.* They are not the only parties it concerns; it occupies a much wider field, and embraces interests and relations of much deeper import. To discuss this question in its true spirit, and to reach all its consequences, it must be examined in all its bearings. Results without this course must be invalid. It is this broad view that governed the decisions of this Association at its former meetings, and its re-affirmance of them at its present session. On this question it was regarded that the *public*, the *science*, and the *profession*, had interests as deeply involved as the students and the schools; and that the Association was bound to look to and protect them."

We must close our hasty notice of this part of the Transactions by expressing the hope that all our readers will procure the volume and read it for themselves. N. S. D.

